# Exploring depression in adolescents: How depression changes in early onset psychosis (EOP), clinical high risk (UHR) and clinical control (CC) patients

**DOI:** 10.1192/j.eurpsy.2021.253

**Published:** 2021-08-13

**Authors:** E. Monducci, G. Colafrancesco, A. Masillo, M. Brandizzi, P. Fiori Nastro, M. Ferrara

**Affiliations:** 1 Department Of Human Neurosciences, University of Rome Sapienza, Rome, Italy; 2 Department Of Mental Health, ASL ROMA1, Rome, Italy; 3 Human Neurosciences, University of Rome, Rome, Italy; 4 Department Of Human Neuroscience, Section Of Child And Adolescent Neuropsychiatry, Sapienza University of Rome, Rome, Italy

**Keywords:** Depression, Calgary depression Scale for Schizophrenia, adolescent, psychosis

## Abstract

**Introduction:**

Depression is very common in adolescent patients and impacts on their quality of life and functioning. Indeed, depression is an important clinical aspect for treatment, outcome, and prognosis.

**Objectives:**

This pilot study investigated the factorial structure of the Calgary depression scale for schizophrenia (CDSS) in a sample of help seeking adolescent patients, stratified in three clinical diagnostic subgroups: early onset psychosis (EOP), clinical high risk (UHR) and clinical control (CC). The relationships between these factors and SIPS domains and subjective experiences were also explored.

**Methods:**

Sixty-nine subjects were examined to assess the severity of depressive symptoms and the degree of subjectively felt cognitive-affective vulnerability (i.e. basic symptoms)

**Results:**

Principal component analysis revealed CDSS to include two main factors, namely: “guilty idea of reference-pathological guilt” (factor I), “depression-hopelessness” (factor II). Two factors revealed multiple correlations with SIPS domains and subjective experiences.

**Conclusions:**

The results confirm the dual factorial structure of CDSS previously reported in the literature in adult samples, further increase our knowledge of the psychopathological components of depression in adolescents, and strongly suggest that CDSS can also be used in early diagnostic settings

**Disclosure:**

No significant relationships.

